# Probing and Enhancing Ligand-Mediated Active Targeting of Tumors Using Sub-5 nm Ultrafine Iron Oxide Nanoparticles

**DOI:** 10.7150/thno.39560

**Published:** 2020-01-22

**Authors:** Yaolin Xu, Hui Wu, Jing Huang, Weiping Qian, Deborah E. Martinson, Bing Ji, Yuancheng Li, Yongqiang A. Wang, Lily Yang, Hui Mao

**Affiliations:** 1 Department of Radiology and Imaging Sciences, Emory University School of Medicine, Atlanta, Georgia, USA.; 2 Boston Children's Hospital, Harvard Medical School, Boston, Massachusetts, USA.; 3 Department of Surgery, Emory University School of Medicine, Atlanta, Georgia, USA.; 4 Integrated Cellular Imaging Core, Emory University, Atlanta, Georgia, USA.; 5 Ocean Nanotech, LLC, San Diego, California, USA.

**Keywords:** Iron oxide nanoparticles, Active targeting, Enhanced permeability and retention, Molecular imaging, Drug delivery.

## Abstract

**Rationale**: “Active targeting” based on the ligand-target affinity is a common strategy to precisely deliver nanoparticle (NP) imaging probes or drug carriers to the diseased tissue. However, such ligand-mediated active targeting inevitably takes place with prerequisite “passive targeting”, driven by the enhanced permeability and retention (EPR) effect. Thus, the efficiency of active targeting in relation to off-targeted unbound NPs is of great importance in quantitative imaging of tumor biomarkers and delivery. With the notion that easy clearance of off-targeted uIONPs may lead to enhanced active targeting and tumor accumulation, we examined the NP size effect on “active targeting” of the transferrin receptor (TfR) using transferrin (Tf)-conjugated sub-5 nm (3 nm core) ultrafine iron oxide NPs (uIONPs) and larger IONPs (30 nm core).

**Methods**: Green fluorescent dye (FITC)-labeled active targeting uIONPs (FITC-Tf-uIONPs) and red fluorescent dye (TRITC)-labeled passive targeting uIONPs (TRITC-uIONPs) were prepared. FITC-Tf-IONPs and TRITC-IONPs were used as comparison for the NP size effect. Multiphoton imaging, confocal fluorescence imaging, histological staining and computational analysis were applied to track different types of NPs in tumors at 1, 3 and 24 hours after co-injection of equal amounts of paired NPs, e.g., active targeting FITC-Tf-uIONPs and non-targeting TRITC-uIONPs, or FITC-Tf-IONPs and TRITC-IONPs into the same mice bearing 4T1 mouse mammary tumors.

**Results**: Active targeting uIONPs exhibited an almost 6-fold higher level of tumor retention with deeper penetration comparing to non-targeting uIONPs at 24 hours after co-injection. However, accumulation of active targeting IONPs with a 30-nm core is only about 1.15-fold higher than non-targeting IONPs. The enhanced active targeting by uIONPs can be attributed to the size dependent clearance of unbound off-targeted NPs, as majority off-targeted uIONPs were readily cleared from the tumor by intravasation back into tumor blood vessels likely due to high interstitial pressure, even though they are not favorable for macrophage uptake.

**Conclusion**: Ligand-mediated active targeting improves the delivery and accumulation of the sub-5 nm NPs. The improvement on active targeting is size-dependent and facilitated by NPs with sub-5 nm core sizes. Thus, sub-5 nm NPs may serve as favorable platforms for development of NP-based molecular imaging probes and targeted drug carriers.

## Introduction

Nanoparticles (NPs) have been widely used as molecular imaging probes and image-guided drug delivery systems, especially in cancer theranostics 1-3. Substantial efforts and advancements have been made in engineering NPs to improve imaging capabilities 4-5, therapeutic efficacy 6, biodistribution, pharmacokinetics 7, and tumor targeting efficiency 8-9. Various cell receptors that are over expressed in tumors are explored as biomarkers for targeted imaging and drug delivery with development of high affinity targeting ligands coupled on the selected NPs. Biomarker specific “active targeting” based on the ligand-target affinity was a common strategy in order to precisely deliver NP imaging probes or drug carriers to tumors after systemic administration 10-12. Ideally, the biomarker specific active targeting should enable quantitatively imaging biomarkers with ligand functionalized NP probes for diagnosis, monitoring disease progression and treatment responses, and directing therapeutics to the targeted disease tissue. However, such ligand-mediated active targeting is inevitably taking place with prerequisite “passive targeting”, driven by the enhanced permeability and retention (EPR) effect that is mediated by leaky tumor vasculature and dysfunctional lymphatic drainage. In fact, it is generally considered that the EPR driven passive targeting plays a dominating role over active targeting, leading to the questions whether the active targeting strategy is sufficiently effective or even necessary. Studies using various tumor targeting ligands, *e.g.,* folic acid 13, amino terminal fragment (ATF) peptide 14, RGD 15, transferrin (Tf) 16, and anti-HER2 antibodies 17, in different animal tumor models showed that active targeting did promote rapid and early binding of NPs to tumor vessels, although the reports on long-term tumor accumulation of NPs were inconsistent. More recently, it is reported that active targeting of NPs only contributes to a minimal amount of cancer cell specific NP uptake in the solid tumors 18. On the other hand, a considerable number of publications suggested the benefit of using ligand-mediated active targeting approaches, which led to better outcomes in tumor delivery and retention of theranostic NPs 13, 19-21. For example, Tf-modified gold NPs presented Tf content-dependent intracellular NP localization in solid tumors 16. More importantly, the presence of EPR-mediated passive targeting and accumulation of off-targeting NPs lead to intrinsic “noise” background that interferes quantitative imaging of biomarkers and delivery of biomarker targeting NPs. Achieving a high level of active targeting to enhance “signal-to-noise ratio” is essential to address this key requirement for targeted therapy by precision medicine.

**Scheme 1:**
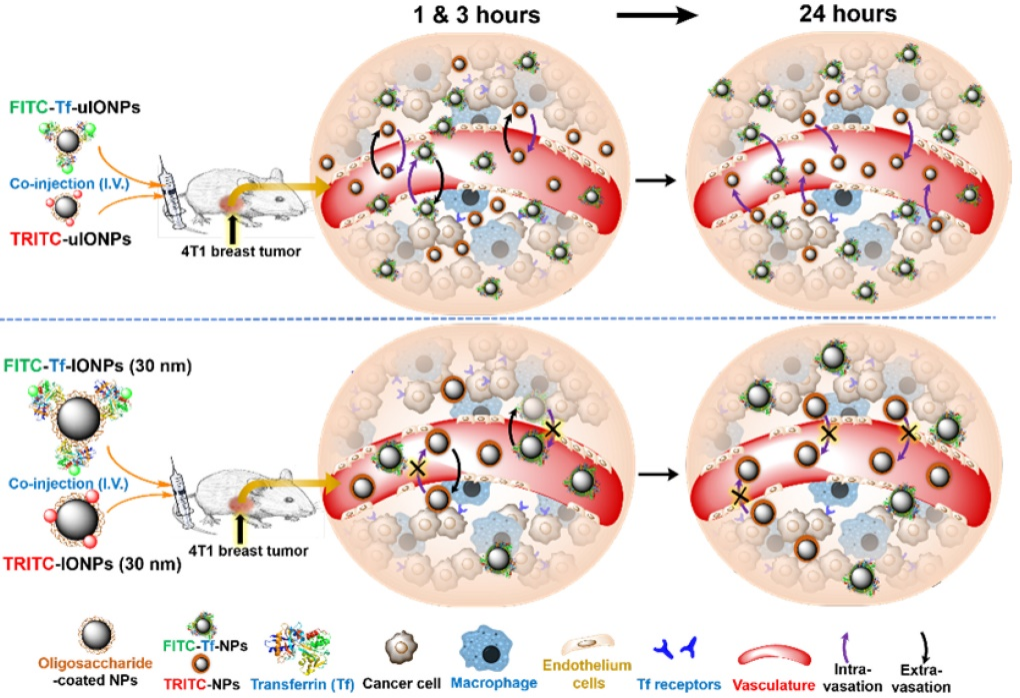
Schematic illustration of the possible mechanism of sub-5 nm uIONPs promoting active targeting that can be tested: At the early time points (1 and 3 hours) after co-injection, both ligand mediated active targeting and passive targeting uIONPs undergo fast extravasation into tumors from the leaky tumor vessels comparing to larger IONPs due to size advantage in permeation; At the late time point (24 hours), TfR targeting uIONPs, *i.e.*, FITC-Tf-uIONPs, may exhibit longer tumor retention and intratumoral distribution within tumor environments due to the specific ligand-target interactions, while the majority of passive targeting uIONPs, *i.e.*, TRITC-uIONPs, as well as other unbound or off-targeted FITC-Tf-uIONPs are cleared out of the tumor and intravasated back into blood vessels but not uptaken by macrophages that prefer larger NPs; However, both ligand mediated active targeting and passive targeting IONPs (30 nm core size) have similar tumoral accumulation due to the limited intravasation.

Noticeably, most of the earlier studies investigated NPs with core sizes of 10-300 nm which are favorable for EPR-driven passive targeting 22-23. In this case, limited intravasation of large sized NPs back into the blood circulation leads to the retention of NPs in the tumor tissue without the need of ligand-mediated active targeting 24-25. A systematic comparison of the size effect on active and passive targeting using spherical gold NPs with core sizes of 15, 30 and 100 nm revealed no significant difference in tumor accumulation of these active and passive targeting gold NPs of different sizes 26. However, NPs with the core sizes smaller than 10 nm have been shown capable of crossing tumor blood vessels and diffusing within interstitial space of tumor tissue with less restrains than their larger counterparts 27-28, and moreover, targeting moieties could also facilitate such smaller NPs to retain within tumor interstitium 29-31. Recently, we have demonstrated that sub-5 nm ultrafine iron oxide NPs (uIONPs) with a core size of 3 nm and unique T_1_-T_2_ dual contrast effect on magnetic resonance imaging (MRI) could exert the EPR effect and enhance intratumoral distribution of NPs compared to larger counterparts due to easier extravasation from the tumor blood vessels and deeper tissue penetration 32. In this work, we rationalize that the efficiency of clearing unbound off-targeted NPs with or without ligand conjugated is important to obtain a high percentage of active targeting in relation to passive targeting. The clearance of unbound off-targeted “by stander” NPs is likely caused by: 1) diffusing away from tumor interstitial space and then intravasation through leaky tumor blood vessels back to circulation or through poor lymphatic vessels; 2) uptake by active macrophages in the tumors. Therefore, uIONPs with a favorable size for clearance would allow for improving active targeting by reducing accumulation of off-targeted NPs. Scheme 1 illustrates the experiments designed to investigate this possible mechanism.

We selected Tf as the tumor targeting ligand because of its specific interactions with Tf receptor (TfR) over-expressed cancer cells and broad applications in receptor-mediated delivery of drug-conjugates 33-34 and tumor imaging 35-36. Multiphoton imaging was used to track fluorescent dye-labeled active targeting-uIONPs and non-targeting uIONPs at the different time points (1, 3 and 24 hours) after co-injecting paired two types of NPs into the same mice bearing orthotropic 4T1 mouse mammary tumors. Results showed substantial differences between time-dependent tumoral accumulation profiles of active and passive targeting uIONPs based on the quantitative analysis of different NPs delivered and accumulated in the tumors.

## Results and Discussion

### Specificity of active targeting uIONPs

**Figure 1:**
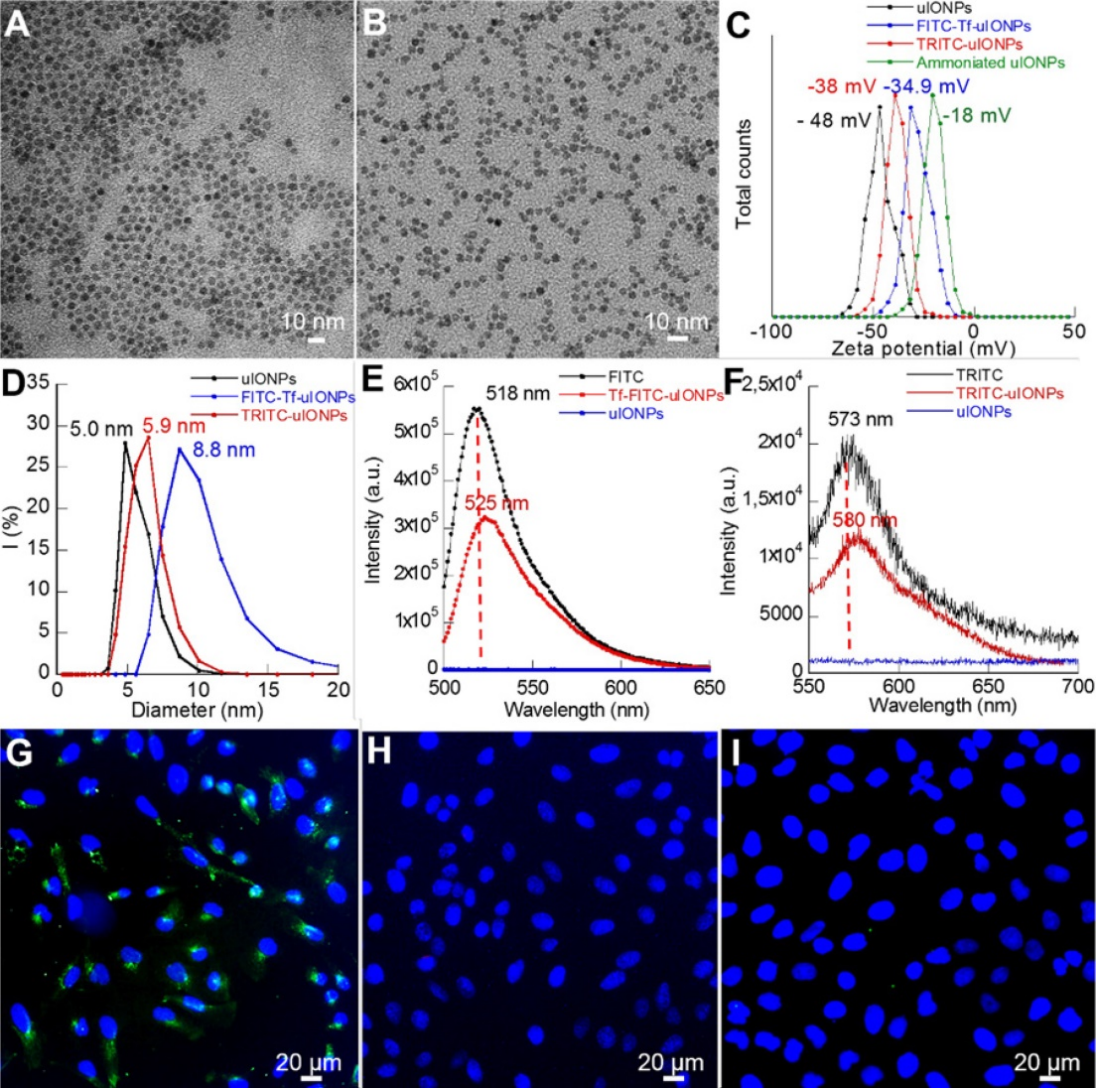
TEM images of (**A**) TRITC-uIONPs, and (**B**) FITC-Tf-uIONPs; DLS (**C**), zeta potential measurements (**D**), and fluorescent emission spectra (**E**-**F**) of uIONPs; confocal fluorescence images of breast cancer 4T1 cells treated with (**G**) targeted FITC-Tf-uIONPs, (**H**) non-targeted TRITC-uIONPs, and (**I**) free Tf and FITC-Tf-uIONPs for the blocking experiment.

To use optical imaging methods to track and evaluate different uIONPs and IONPs in real-time for the role of passive and active targeting on tumoral delivery of NPs, tumor-targeting uIONPs labeled with a green fluorescent dye (FITC-Tf-uIONPs) and non-targeting uIONPs labeled with a red fluorescent dye (TRITC-uIONPs) were first prepared. Both FITC-Tf-uIONPs and TRITC-uIONPs were evaluated for their physicochemical properties at the completion of the preparation and before each set of *in vitro* and *in vivo* experiments. As shown in Figure 1A-B, both FITC-Tf-uIONPs and TRITC-uIONPs were stable and monodispersed with a fairly uniform core size of 3 nm as measured by transmission electric microscopy (TEM), and showed a tight size distribution range in the dynamic light scattering (DLS) measurement with no statistically significant change after storage at 4 °C for one or two weeks. Zeta potential measurements revealed the reduction of negative surface charge (from -48 to -18 mV) as the result of successful ammonization of the oligosaccharide coating to introduce -NH_2_ functional groups on the surface. After reacting with FITC-Tf or TRITC, zeta potentials of FITC-Tf-uIONPs and TRITC-uIONPs changed to -35 and -38 mV (Figure 1C), respectively, indicating successful conjugation of the ligands and/or dyes 40-41. Compared to the hydrodynamic size of oligosaccharide-coated uIONPs measured by DLS, the size of TRITC-uIONPs was nearly unchanged, and the slight hydrodynamic size increase (~ 4 nm) on FITC-Tf-uIONPs due to conjugation of Tf ligands onto uIONP surfaces (Figure 1D). The presence of small red-shift and reduced intensity in the emission spectra of FITC-Tf-uIONPs and TRITC-uIONPs (Figure 1E and F) compared to that of free FITC or TRITC was attributed to the weak “quenching” effect on dyes due to the existence of uIONPs, which presented strong absorption in the visible range 42-43. The ligand density was estimated as 3 Tf ligands per uIONP (14 Tf ligands per IONP) based on the result from the bicinchoninic acid assay (BCA) for quantifying proteins 44.

To evaluate the targeting property of FITC-Tf-uIONPs and TRITC-uIONPs, cell binding assays were performed *in vitro* on the 4T1 mouse mammary tumor cell line over-expressing TfR. As shown in Figure 1G, sharp green fluorescence around cells indicated that a high level of FITC-Tf-uIONPs bound to 4T1 cells, but no visible red fluorescence was observed from the cells treated with non-targeting TRITC-uIONPs (Figure 1H). To further confirm that the Tf ligand was responsible for the specific recognition of FITC-Tf-uIONPs by 4T1 cells, a blocking experiment was conducted by first treating 4T1 cells with free Tf and then FITC-Tf-uIONPs. As shown in Figure 1I, we observed very limited uptake of ligand conjugated FITC-Tf-uIONPs by 4T1 cells with undetectable green fluorescence. The cell viability tests with various concentrations (0-250 μg Fe/mL) of FITC-Tf-uIONPs and TRITC-uIONPs indicated that no significant toxicity from uIONPs (Figure S1A-B).

We further validated the targeting specificity with *ex vivo* imaging analysis of the 4T1 tumor tissue treated or co-stained with both FITC-Tf-uIONPs and TRITC-uIONPs. The 4T1 tumor model used in this study was confirmed to overexpress TfR as demonstrated by the immunohistochemistry (IHC) assay using FITC-labeled Tf against TfR (Figure S2). Specifically important for this study, using the intact tumor tissue samples with tissue cytoarchitecture and intercellular connections mimicking *in vivo* conditions 45-46 to test the specificity of the probe provided evidences for: (1) confirming the presence and over expression of TfR in the 4T1 mouse mammary tumors in mice subsequently used for *in vivo* NP delivery experiments, and (2) validating ligand mediated active targeting FITC-Tf-uIONPs in the tumor tissue that retains morphological structure, heterogeneous cell types and population as well as tumor microenvironment, not just the homogenous cell culture condition. Tumor tissue sections co-stained with FITC-Tf-uIONPs and TRITC-uIONPs exhibited a much higher green fluorescence intensity from FITC-Tf-uIONPs with a more spread-distribution pattern (Figure S3) comparing to the weak red fluorescence from TRITC-uIONPs (Figure S3B-C) under the same co-staining conditions. Combined with observations in confocal fluorescence images and H&E staining for the tumor morphological structure (Figure S3E), it was found that tumor-targeting FITC-Tf-uIONPs located at the tumor center, which consisted of TfR over-expressed tumor cells. In comparison, non-targeting TRITC-uIONPs mainly resided in the stroma areas around the tumor nest (circled with dashed red curves) 47-48. The observations from analysis of intratumoral distributions of different NPs indicated that FITC-Tf-uIONPs were capable of targeting 4T1 tumors through interactions of Tf ligands conjugated on NPs to the targeted TfR. To rule out the possible contamination of the free fluorescent dyes or auto-fluorescence from the tissue, Prussian blue staining for iron was performed to examine the presence of uIONPs on the same tumor slice used for confocal fluorescence imaging. The strong blue stain could be found to co-localize with green fluorescence from FITC-Tf-uIONPs (Figure S3F). In addition, the differences in the distribution of stained blue color between the tumor regions with dense tumor cells and peripheral areas further suggested that ligand-target mediated binding between tumor cells and FITC-Tf-uIONPs led to higher accumulation of FITC-Tf-uIONPs on the heterogeneous tumor tissue.

### Comparison of tumor uptake of active targeting and non-targeting uIONPs

To examine the impact of ligand mediated active targeting on uIONP delivery and retention in tumors by directly comparing the ligand conjugated NPs with the same NPs without ligands, an equal amount of non-targeting TRITC-uIONPs (10 mg Fe/kg) and active targeting FITC-Tf-uIONPs (10 mg Fe/kg) were mixed and then co-injected into the same 4T1 tumor-bearing mice. Comparing to conventional approaches of comparing different agents that need to perform the experiments with different groups of animals, the approach of co-injection of two pairing agents in the same animal ensures the same delivery route and physiological environment that both agents navigate through, effectively minimizing, if not eliminating, the inter-subject variations. Since different NPs paired for comparison are labeled with two wavelength distinctive fluorescent dyes, multiphoton imaging can be used to track the location and amount of each type of NPs in the same region of the tumor for direct comparison of their distribution and accumulation. In this study, time dependent observations and measurements of fluorescent dye-labeled active targeting and non-targeting uIONPs were carried out at 1, 3 and 24 hours after co-injection to differentiate and examine the differences in dynamic processes of targeted delivery and clearance of unbound off-targeted FITC-Tf-uIONPs and TRITC-uIONPs in the same tumors.

**Figure 2:**
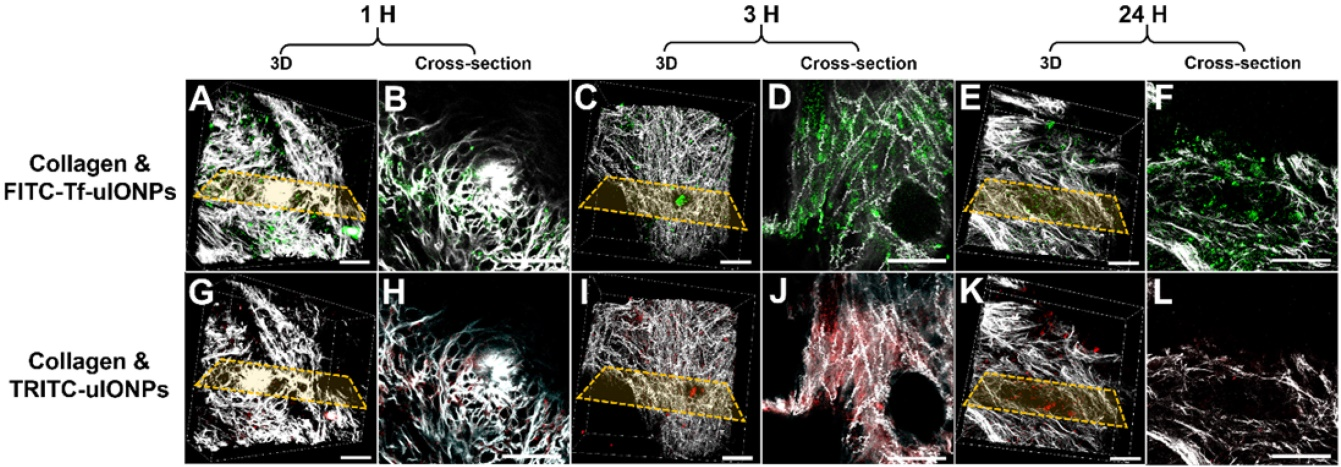
3D re-construction of multiphoton microscopic images taken from an 8 mm^3^ tumor tissue block collected from 4T1 tumor-bearing mice co-injected with active targeting FITC-Tf-uIONPs (green) and non-targeting TRITC-uIONPs (red) at different time points (**A** and **G** for 1 hour, **C** and **I** for 3 hours, **E** and **K** for 24 hours after injection) with the selected cross-sections (**B** and **H** for 1 hour, **D** and **J** for 3 hours, **F** and **L** for 24 hours). Tumor collagen was visualized using second harmonic generation (SHG), and presented as bright signals in a grayscale setting. The scale bar for all images is 50 µm.

As shown in multiphoton microscopy images of selected tumor slices, co-injected active targeting FITC-Tf-uIONPs with green fluorescence and non-targeting TRITC-uIONPs with red fluorescence exhibited similar tumor uptake and intratumoral distributions at the time points of 1 hour (Figure 2B *vs.* 2H) and 3 hours (Figure 2D *vs.* 2J) after co-injection. Observed similar level of NP delivery and intratumoral distributions suggests that at the early time points, NP delivery was predominately due to the passive targeting, mostly driven by the EPR effect as reported in our previous study on improved intratumoral delivery of sub-5 nm sized uIONPs 32. However, 24 hours after co-injection, differences in the amounts of active targeting FITC-Tf-uIONPs and non-targeting TRITC-uIONPs delivered to the tumor and their intratumoral distributions became significant, revealing the distinct tumor retention properties of two different types of uIONPs (Figure 2F-L). While the red fluorescent signal from non-targeting TRITC-uIONPs markedly faded away, the active targeting FITC-Tf-uIONPs remained in the tumor region, indicated by a noticeably higher level of green fluorescence from FITC-Tf-uIONPs comparing to red fluorescence from TRITC-uIONPs. We then used 3D re-construction of multiphoton microscopic images from all scanned tumor slices to determine the penetration depth and intratumoral distribution profiles of both active and passive targeting uIONPs within an 8 mm^3^ tumor block. Similar to the time dependent retention behaviors observed in 2D multiphoton images, the 3D presentation of the results further demonstrated the substantially different profiles of tumoral accumulation and clearance of active targeting FITC-Tf-uIONPs (Figure 2A, 2C and 2E) and non-targeting TRITC-uIONPs (Figure 2G, 2I and 2K) in the same tumor regions.

**Figure 3:**
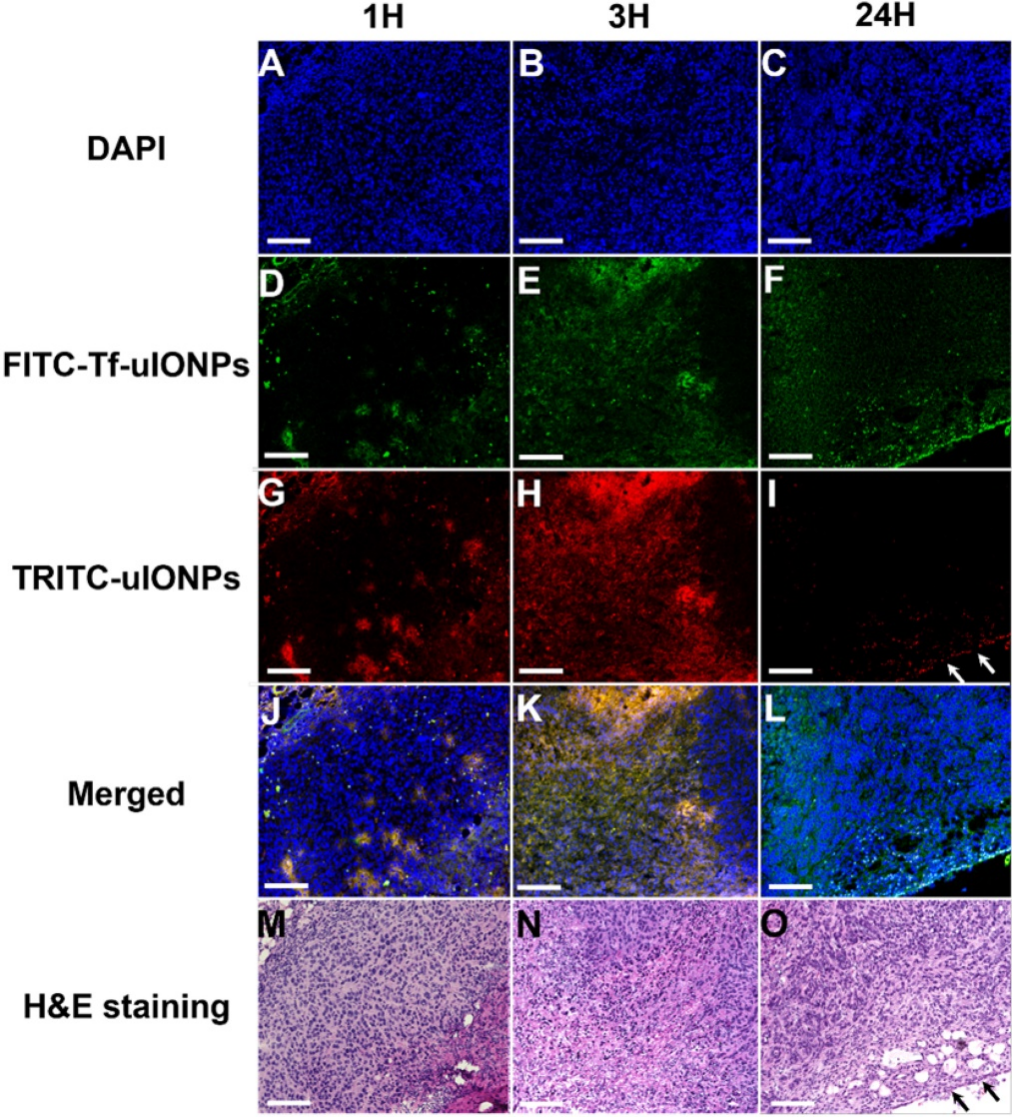
Confocal microscopic images of DAPI stained nuclei (**A**, **B**, **C**), FITC-Tf-uIONPs (**D**, **E**, **F**), TRITC-uIONPs (**G**, **H**, **I**), merged images (**J**, **K**, **L**), and H&E staining (**M**, **N**, **O**) of tumor sections from 4T1 tumors collected at different time points (1, 3 and 24 hours) after tumor bearing mice receiving co-injection of FITC-Tf-uIONPs and TRITC-uIONPs. The arrows indicate the edge of the tumor. The scale bar for all images is 200 µm.

The time dependent intratumorial distribution, retention and clearance of uIONPs observed in multiphoton imaging studies were further validated with confocal fluorescence imaging and histological (H&E) staining of tumor sections of the collected tumors. Within 3 hours after co-injection of active targeting FITC-Tf-uIONPs and passive targeting TRITC-uIONPs, both types of uIONPs were co-localized mostly in the same regions of the tumor with no significant difference in the amount of uptake based on the fluorescence intensities measured in different channels (Figure 3D-F *vs.* Figure 3G-I). To quantitatively assess the delivery amount and intratumoral distribution of active targeting and passive targeting uIONPs at different time points after co-injection, we quantified the pixel values of ligand conjugated FITC-Tf-uIONPs and non-specific TRITC-uIONPs based on different fluorescence signals of dye labeled uIONPs. The pixels of different uIONPs from the same region of interest in multiple confocal microscopic images of randomly selected tumor slices were segmented. The pixel values of FITC-Tf-uIONPs over TRITC-uIONPs were calculated to show the difference of their intratumoral accumulation levels. All ratios of pixel values from active targeting ligand conjugated uIONPs *vs.* non-targeting uIONPs in the selected fields of view were around 1 in the early times (*i.e.*, 1 and 3 hours) after co-injection (Figure S4).

In contrast to the distribution patterns observed at 1 and 3 hours after co-injection, active targeting FITC-Tf-uIONPs appeared to be more spread into the tumor tissue 24 hours after co-injection, evidenced by observing increased green fluorescence signal from FITC. In comparison, the level of red fluorescence signal from passive targeting TRITC-uIONPs was significantly reduced within the same slices and mostly spotted at the edge of the tumor, as shown in the confocal microscopic and H&E stained images (Figure 3I and O). These results suggest continuous intratumoral accumulation and little clearance of active targeting FITC-Tf-uIONPs at the later time point (24 hours after injection), comparing to passive targeting uIONPs which were found mostly in the tumor edge, a pattern likely resulted from low intravasation or clearance from the tumor via the blood vessels due to a high interstitial pressure 49-50. This substantial difference in intratumoral accumulation between active targeting and non-targeting uIONPs was confirmed by measuring the pixel ratios of FITC-Tf-uIONPs over TRITC-uIONPs, which significantly increased from 1.3, which is a maximum value of the ratio within 3 hours of co-injection of uIONPs, to 6.9 (a maximum value of the ratio among the slices measured) at 24 hours after co-injection of uIONPs (Figure S4r).

Taking all results together, at the late time point, *i.e.,* 24 hours after co-injection of two types of uIONPs, active targeting, mediated by the Tf-TfR or ligand-target interactions, became pronounced, leading to increased retention of actively targeted FITC-Tf-uIONPs. However, most passively targeted or unbound off-targeted uIONPs were likely expelled from the tumors due to easy clearance of non-targeting uIONPs that lack binding affinity between uIONPs and tumor cells. Thus, multiphoton and confocal fluorescence imaging, assisted by quantitative image analysis (with 3D rendering), revealed that the ligand-mediated active targeting plays substantial roles in promoting and enhancing delivery of sub-5 nm uIONPs over EPR-driven passive targeting, leading to high retention of actively targeted uIONPs in the tumors over time.

While the current study specifically focused on the sub-5 nm size-depend effect on the ligand-mediated active targeting, it should be mentioned that surface properties, blood circulation time, blood concentration of NPs, the type of tumor models, specific targets and ligands selected as well as the affinity of the ligand-to-target are among the many factors affecting the complicated *in vivo* NP delivery to the tumors. In addition to the ligand mediated and promoted interactions with the targeted cells, other surface properties, such as hydrophilicity, electrostatic and surface charges, all can contribute to the interactions of nanomaterials with the biological environments varying from blood, tissue interstitial space, cell surface, to the cellular compartment. We purposely designed the experiments to minimize the differences in the surface properties when comparing two paired NPs for *in vivo* tumor delivery and multiphoton imaging studies on active targeting and targeted delivery, using: 1) the same coating material (*i.e.*, oligosaccharides); 2) the same NP core and core size (*i.e.*, uIONP or IONP); 3) the same surface functional groups (*i.e.*, amide); 4) same targeting ligand (*i.e.*, Tf); 5) similar surface potential or charges (*e.g.*, -35 mv for FITC-Tf-uIONPs *vs.* -38 mV for TRITC-uIONPs); and finally, 6) co-injection of two comparing NPs into the same animals. Nevertheless, more thorough and systematic investigations on the effects of surface properties of NPs on the targeted delivery are necessary in the future.

### Effect of NP size on the active targeting facilitated delivery and retention

**Figure 4:**
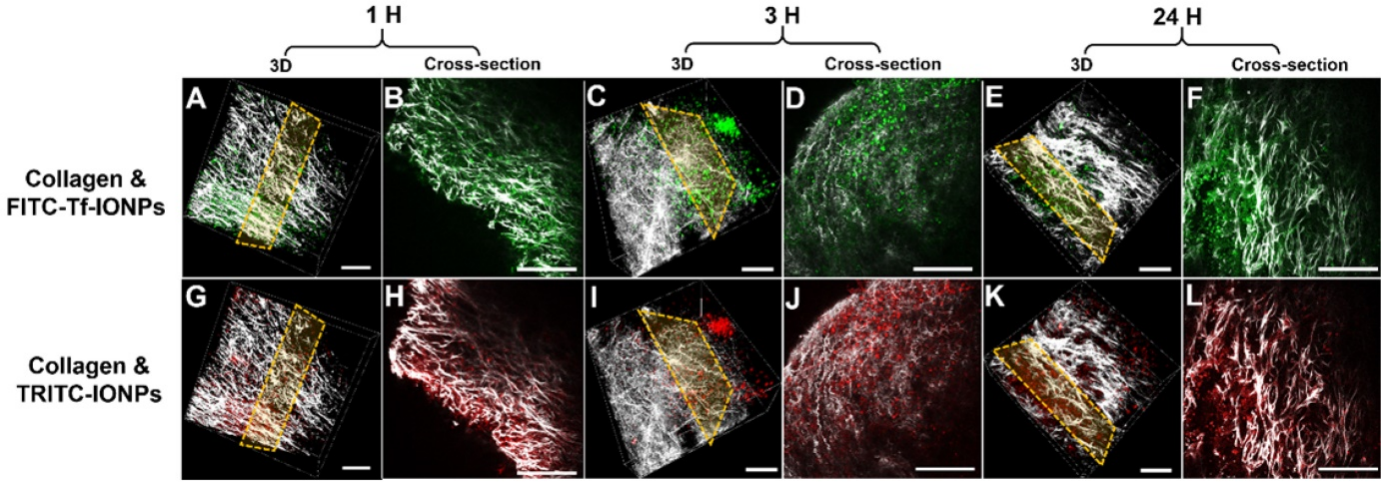
3D re-construction of multiphoton images taken from an 8 mm^3^ 4T1 tumor tissue block collected from tumor-bearing mice co-injected with ligand conjugated active targeting FITC-Tf-IONPs (green) and non-targeting TRITC-IONPs (red) at different time points after injection (**A** and **G** for 1 hour, **C** and **I** for 3 hours, **E** and **K** for 24 hours) with the selected cross-sections (**B** and **H** for 1 hour, **D** and **J** for 3 hours, **F** and **L** for 24 hours). Tumor collagen is shown in gray scale. The scale bar for all images is 50 µm.

To investigate the possible mechanism by which the active targeting facilitates delivery and tumor retention of NPs through promoting clearance of unbound or “by stander” off-targeted NPs (as illustrated in Scheme 1), we further performed the same multiphoton imaging experiments of tracking NP delivery to tumors using IONPs with an averaged core size of 30 nm instead of uIONPs. The time dependent tumor accumulation and retention profiles between FITC labeled active targeting FITC-Tf-IONPs and TRITC labeled passive targeting TRITC-IONPs as well as those of uIONPs were compared based on the quantitative analysis of image data. Unlike sub-5 nm uIONPs, in this case, we anticipated that both passive targeting IONPs with a 30-nm core size and unbound active targeting IONPs may still retain in the tumor even in the later time points due to limited clearance of larger IONPs by intravasation to the tumor micro-vessels. Therefore, IONPs would have a lower ratio of active targeting NPs over passive targeting NPs than that of uIONPs due to lack of clearance of “by stander” unbound off-targeted NPs. For these experiments IONPs were made of same materials as those for uIONPs except for the difference in the core size (shown in Figure S5). After co-injection of the equal amount (*i.e.*, 10 mg Fe/kg) of active and passive targeting IONPs into mice bearing 4T1 breast tumors, tumor tissues were excised and scanned with the multiphoton imager and confocal fluorescence microscopy at the same time points (*i.e.*, 1, 3 and 24 hours) as the experiments done with uIONPs. As shown in cross-section 2D multiphoton images (Figure 4B and 4H for 1 hour, 4D and 4J for 3 hours, and 4F and 4L for 24 hours) and 3D images (Figure 4A and 4G for 1 hour, 4C and 4I for 3 hours, and 4E and 4K for 24 hours), accumulation and retention profiles of active targeting and passive targeting IONPs (a core size of 30 nm) in tumors appear to be at the same level over all three different time points, which is significantly different from the observation of substantial difference in the tumor accumulation between active targeting and passive targeting uIONPs.

**Figure 5:**
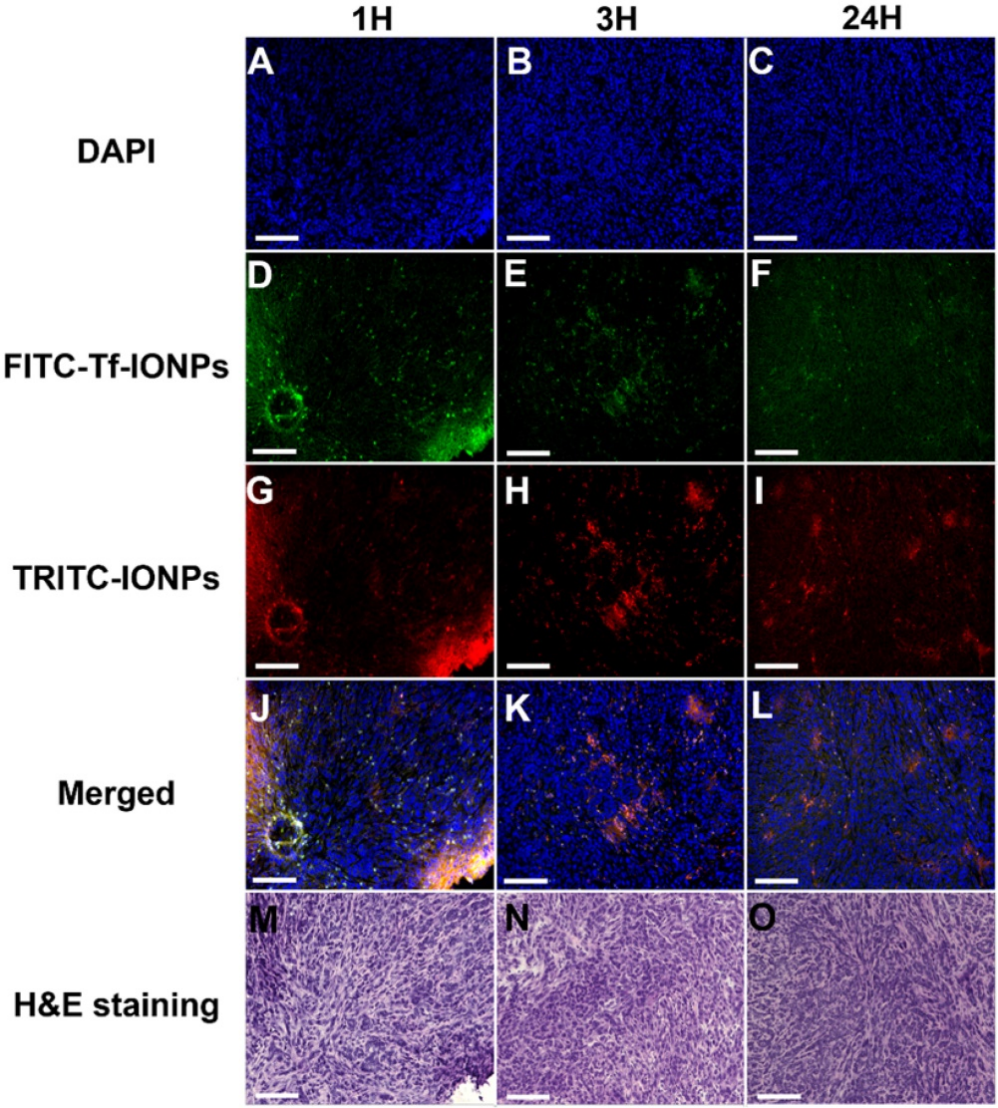
Confocal microscopic images of nuclei (**A**, **B**, **C**), FITC-Tf-IONPs (**D**, **E**, **F**), TRITC-IONPs (**G**, **H**, **I**), merged images (**J**, **K**, **L**), and H&E staining (**M**, **N**, **O**) of tumor sections, collected from 4T1-tumor-bearing mice at different time points (1, 3 and 24 hours) after co-injection of FITC-Tf-IONPs and TRITC-IONPs. The scale bar for all images is 200 µm.

Subsequently, results from multiphoton imaging of accumulation and retention of 30 nm IONPs in 4T1 tumors were validated with confocal fluorescent imaging and H&E staining of the tumor sections of the same tumors. At three different time points of the study, no significant difference in tumor uptake of tumor-targeting and non-targeting IONPs was observed from confocal microscopic images as shown in Figure 5. Correspondingly, the pixel ratios (active over passive targeting IONPs) at different time points (Figure S6) were fluctuating at 1.23 ± 0.25, further confirming the similar tumor accumulation profiles between active targeting FITC-Tf-IONPs and passive targeting TRITC-IONPs. In this case, larger IONPs with a 30-nm core size may hardly escape from the tumor environment, and active targeting could only slightly enhance (max at 0.5 times) the intratumoral delivery of IONPs comparing to the EPR driven passive targeting.

**Figure 6:**
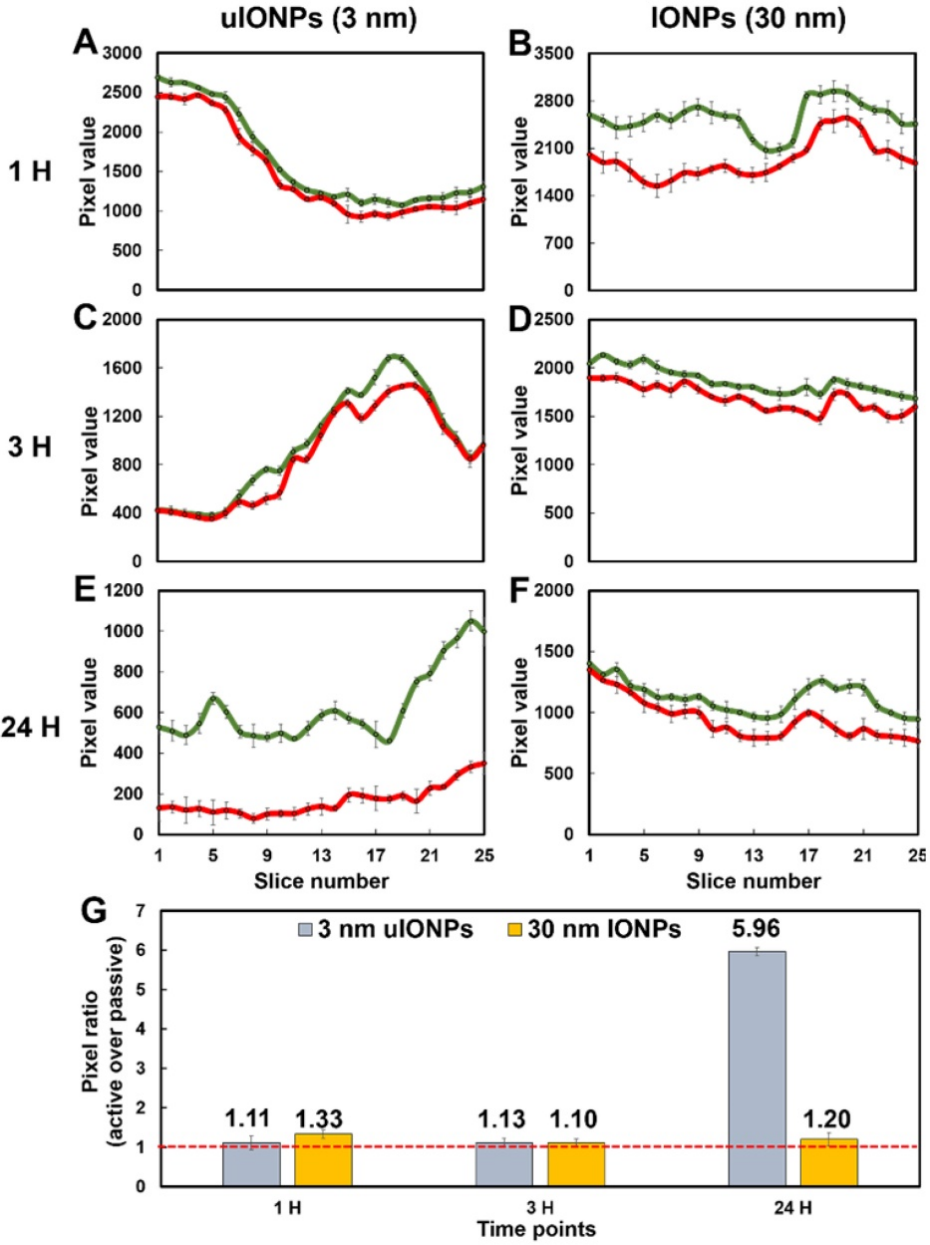
Plots of levels of the tumor delivery and accumulation of different NPs in the selected tumor sections at different time points. Fluorescence signals from dye-labeled NPs were measured from a series of multiphoton images of a tumor block (25 slices and 2 μm thick/slice) and counted (presented in the pixel value). Images were collected at various time points (1, 3, and 24 hours) after co-injection of ligand conjugated active targeting FITC-Tf-uIONPs with a 3-nm core size (green curves) and passive targeting TRITC-uIONPs (red curves) (**A**, **C**, and **E**), or active targeting FITC-Tf- IONPs with a 30 nm core size (green curves) and passive targeting TRITC-IONPs (red curves) (**B**, **D** and **F**). The pixel ratios of active targeting NPs over passive targeting NPs were calculated and averaged from the data measured from 25 selected slices (**G**). The dashed red line indicated the pixel ratio at 1.

To further elucidate the size effect of NPs on the active targeting, we compared the time-dependent intratumoral accumulation profiles of active targeting and passive targeting uIONPs and larger IONPs based on the ratios of pixel values of paired active targeting and passive targeting NPs detected in the same tumor tissue sections. Figure 6 presents the plots of pixel values of each type of NPs measured in the sequentially cut tumor slices (25 slices and 2 μm thick/slice) at different time points after co-injection of paired comparing NPs. Paired active targeting and passive targeting uIONPs with a 3 nm core size (Figure 6A, 6C) or active targeting and passive targeting IONPs with a 30-nm core size (Figure 6B, 6D) exhibited mostly similar delivery and accumulation levels in each tumor section at the early time points (1 and 3 hours) after injection. However, at the time point of 24-hour after co-injection, the levels of active targeting uIONPs became higher than that of the passive targeting uIONPs in all measured tumor sections (Figure 6E), while the levels of active targeting and passive targeting IONPs with larger score sizes remained similar (Figure 6F). When averaging the levels of active and passive targeting NPs from all slices at each delivery time point, the ratios of active and passive targeting NPs, as plotted in Figure 6G, are around 1 in the early time points of delivery of either uIONPs or larger IONPs, regardless of active targeting or passive targeting or size difference. Therefore, there is no difference in active and passive targeting in the early delivery time point, as the EPR driven passive targeting likely played the predominant role in NP delivery initially. However, the ratios of active and passive targeting uIONPs increased from 1.13 at 3 hours after co-injection to 5.96 at 24 hours after co-injection (Figure 6G). In contrast, the ratios of active targeting over passive targeting of larger 30-nm core IONPs at 24 hours remained nearly unchanged, *i.e.*, from 1.33 at 1 hour after co-injection to 1.20 at 24 hours after co-injection with no statistically significant difference. Thus, sub-5 nm uIONPs clearly exhibited a size-dependent advantage in promoting ligand mediated active targeting in delivery and accumulation of NPs in the tumors. Importantly, the increased ratios of active targeting uIONPs over passive targeting uIONPs in the later time points of delivery, *e.g.*, 24 hours after co-injection, is largely due to approximately 50% reduction of pixel values of passive targeting uIONPs rather than the increase of active targeting uIONPs based on the pixel counts.

Taking these observations together, it can be postulated that the increased level of actively targeted delivery and accumulation is likely attributed to the more clearance of non-targeting NPs from the tumors. Interestingly, comparing to uIONPs, larger sized IONPs appear to have the higher level of total delivery and accumulation in tumors but with little difference in delivery of actively and passively targeted NPs, even at 24 hours after co-injection. This size-dependent tumor retention difference at 24 hours after injection supports the proposed mechanism describing the NP size effect on the active targeting and tumor retention of NPs, in which easy clearance of unbound off-targeted uIONPs but not those bound on the tumor cells due to ligand-receptor affinity is the factor to facilitate ligand-mediated intratumoral retention of the active targeting IONPs.

It should be noted that delivery of NPs to tumors is significantly affected by their circulation or blood half-time which is strongly dependent on the NP size 51-52. The prolonged blood circulation half-time is crucial for increased tumor accumulation 53. Moreover, the permeability of NP into the tumors can be benefited from the long blood circulation half-time with the favorable EPR effect at a higher blood concentration of NPs 54. Larger NPs typically have shorter blood circulation half-times due to higher and fast uptake of larger NPs by reticuloendothelial system (RES). The blood circulation time of reported uIONPs was estimated at 10 hours based on our previous studies 32. Therefore, sub-5 nm uIONPs also take advantages of other size-dependent effects in the EPR driven delivery as demonstrated in the early report 32.

**Figure 7:**
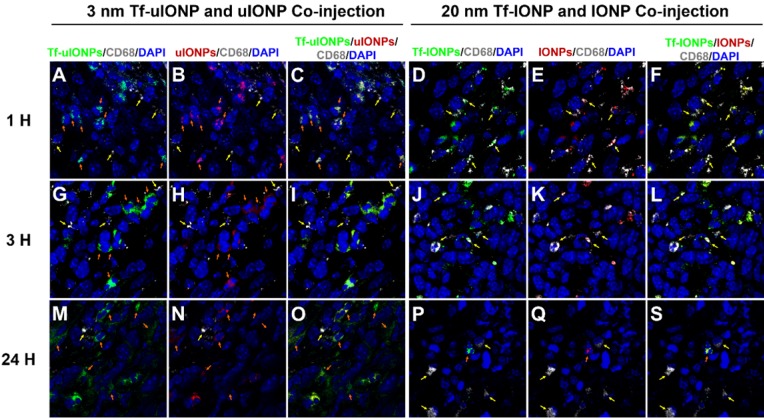
Confocal fluorescence images of 4T1 breast tumor tissue sections collected at 1 (**A** to **C**), 3 (**G** to **I**) and 24 (**M** to **O**) hours after i.v. co-injection of active targeting FITC-Tf-uIONPs (green) and passive targeting TRITC-uIONPs (red) with comparison of images collected at 1 (**D** to **F**), 3 (**J** to **L**) and 24 (**P** to **S**) hours after i.v. co-injection of 30 nm-core active targeting FITC-Tf-IONPs (green) and non-targeting TRITC-IONPs (red). There is little overlapping of FITC-Tf-uIONPs or TRITC-uIONPs (indicated by orange arrows) and CD68^+^ macrophages (indicated by yellow arrows) at different time points. In comparison, a small amount of macrophages were found co-localized with 30 nm IONPs (indicated by yellow arrows) in the tumor tissue sections collected at 1 and 3 hours after the co-injection. Macrophages were stained by anti-CD68 antibody with fluor647 tagging (white), and nuclei were stained by DAPI (blue).

It is reported that tumor associated macrophages (TAMs) are considered as the primary scavenger components in tumors to remove foreign materials, including IONPs, in the size-dependent manner 55. Indeed, macrophages were clearly observed from the 4T1 breast tumor tissue (Figure S7a-b). In order to determine whether the tumoral clearance of passive targeting or unbound off-targeted uIONPs was mainly through uIONPs intravasation back to the tumor blood vessels as the result of high interstitial pressure, or due to macrophage clearance of NPs, we examined the macrophage uptake of different sized FITC-labeled IONPs (*i.e.,* 3, 10, 20, and 30 nm). After treating macrophages (Raw246.7) with FITC-labeled IONPs of different sizes, we observed that macrophages exhibited low uptake of small sized uIONPs with weakest green fluorescence from uIONP-treated macrophages but high uptake of larger IONPs with much stronger green fluorescence from larger-IONP-treated macrophages as shown in Figure S6c-f in the size-dependent manner. To compare the macrophage uptake of different NPs quantitatively, pixels of green fluorescent signals from the fluorescent images of macrophages treated with different FITC-labeled NPs were segmented and quantified. The results on the amount and level of macrophage uptake of different NPs summarized in Figure S6g indicate that macrophage uptake of NPs has the preference for larger NPs, which is consistent with the reports by others 56-57. Using the anti-CD68 antibody against the macrophage marker CD68 58, IHC staining tumor tissue sections collected at different time points after co-injections of paired FITC-Tf-uIONPs and TRITC-uIONPs or paired FITC-Tf-IONPs and TRITC-IONPs showed little overlap of CD68 with FITC-Tf-uIONPs and TRITC-uIONPs in the early time points (*i.e.*, 1-3 hours) after co-injection (Figure 7A-C, and Figure 7G-I), whereas almost no overlap of CD68 with FITC-Tf-uIONPs and TRITC-uIONPs at 24 hours after co-injection (Figure 7M-O). In comparison, as shown in Figure 7D-F, Figure 7J-L, and Figure 7P-S, the overlapping of CD68 with FITC-Tf-IONPs and TRITC-IONPs was noticeably higher in all time points than that of uIONPs. Regardless the presence of CD68^+^ macrophages, in the cases of either uIONPs or IONPs, a significant amount of uIONPs or IONPs was found in the stained tumor tissue sections.

These results suggest that intravasation of passive targeting or unbound off-targeted uIONPs back into the tumor blood vessels is the possible route of their clearance from the tumor, since the uptake of sub-5 nm sized uIONPs by the macrophage is likely minimal comparing to those with larger sizes. Worth noting, reduction or elimination of unbound off-targeted “by stander” NPs that interfere the active targeting as background can be as important as enhancing the delivery of active targeting NPs when considering to quantify targeted biomarkers or targeted delivery. It is likely that when a large sized NP is used, the effect of active targeting can be dampened by the presence of almost equal amount of the off-targeted NPs which are difficult to be cleared from the tumor through tumor blood vessels due to their relatively poor intravasation. Therefore, larger NPs are less favorable for gaining a higher level of active targeting delivery because of restricted intravasation off-targeted NPs back into the blood circulation or clearance. Unbound or passive targeting IONPs lead to the un-wanted background level that reduces the partition of active targeting IONPs, eventually interfering with the quantitation of active targeting IONPs as desired by advanced molecular imaging and drug delivery. While our findings should be viewed with the considerations that tumors are highly heterogeneous, and the preparation and properties of NPs and their targeting ligands vary in different studies, nevertheless this work demonstrated that the rational design of NP-based theranostics utilizing both enhanced EPR effect and efficient active targeting to minimize the non-specific accumulation and background from unbound off-targeted NPs and further improve the ligand-mediated active targeting of biomarkers.

## Conclusions

We have shown that under the same delivery route and tumor microenvironment, the smaller sized IONPs, such as sub-5 nm uIONPs, exhibited a significantly higher level of active targeting delivery than that of passive targeting delivery. There is a 6-fold increase in active targeting facilitated tumor accumulation of uIONPs, compared to that of passive targeting ones. Ligand mediated active targeting led to a time dependent accumulation of uIONPs with deeper tumor penetration and prolonged tumor retention time. The enhanced active targeting by uIONPs can be attributed to the size dependent clearance of unbound off-targeted uIONPs, as majority off-targeted uIONPs were readily cleared from the tumor by intravasation back into tumor blood vessels likely not taken up by macrophages. The phenomenon of the size dependent tumoral clearance of unbound off-targeted NPs is also supported by the evidence derived from comparison of uIONPs with 30 nm IONPs under the same experimental conditions, as the active targeting only showed a marginal advantage than passive targeting in tumoral uptake and accumulation of 30 nm IONPs. These findings further suggest that the ligand mediated active targeting strategy is a valid approach to the targeted delivery of NPs to the tumors under the conditions that can reduce non-specific accumulation of non-targeting or off-targeted NPs. For future development in engineering new NPs for biomarker targeted applications, sub-5 nm NPs, such as uIONPs presented in this work, should be able to offer promising platforms for the development of biomarker quantitative molecular imaging probes and image-guided delivery of NP-based theranostics.

## Materials and Methods

### Synthesis of fluorescent dye-labeled, active targeting and passive targeting uIONPs and IONPs

The preparation of fluorescent dye-labeled, passive targeting uIONPs consisted of three steps. In brief, the oligosaccharide-coated uIONPs with a core size of 3 nm were first synthesized following our previously published method 37. To further label uIONPs with fluorescent dyes, hydroxyl groups of the oligosaccharide coating were partially ammoniated by mixing 1 mg of oligosaccharide-coated uIONPs within 2 mL of ammonia hydroxide solution at 37 °C overnight 32. The ammoniated uIONPs were carefully purified with Amicon Ultra-4 centrifugal filters (50 kDa) for several times to remove excess amines and re-dispersed into sodium bicarbonate buffer (1 mg Fe/mL). Finally, 50 µL of red fluorescent dye, TRITC (1 mg/mL in DMSO, and emission wavelength at 576 nm), was reacted with 1 mg of ammoniated uIONPs for 2 hours at room temperature. The conjugation between TRITC and ammoniated uIONPs were completed based on the specific interactions between -N=C=S groups (on dye molecules) and -NH_2_ groups (on uIONP surfaces). TRITC-uIONPs were purified into de-ionized water (1 mg Fe/mL) and used as non-targeting controls for further studies.

To prepare fluorescent dye-labeled, ligand conjugated active targeting uIONPs, 50 µL of green fluorescent dye, FITC (1 mg/mL in DMSO, and emission wavelength at 525 nm) was first reacted with 1 mg of targeting ligand Tf (1 mg/mL in sodium bicarbonate buffer) for 2 hours. The coupling of FITC to Tf was based on the interactions between -N=C=S groups (on dye molecules) and -NH_2_ groups (on Tf molecules). After Tf-FTIC was collected in the activation buffer (pH = 5.5) with Tf concentration of 1 mg/mL, a mixture of EDC (0.5 mg)/NHS (0.25 mg) was added to the FITC-Tf solution, which was stirred for 0.5 hour to activate the carboxyl groups of Tf for conjugation with amine groups from ammoniated uIONPs. Subsequently, ammoniated uIONPs (1 mg Fe/mL in the coupling buffer) were introduced to the above-mentioned mixture. The reaction for conjugation lasted for 2 hours at room temperature. Finally, FITC-Tf-uIONPs were purified with Amicon Ultra-4 centrifugal filters (50 kDa), re-dispersed into water (1 mg Fe/mL). Similar surface modifications were used for labeling fluorescent dyes and conjugating Tf onto IONPs with the core size of 30 nm.

### Characterizations of ligand conjugated uIONPs and IONPs

The size and morphology of NPs were examined before and after surface functionalization under a transmission electron microscope (TEM, Hitachi H-7500, Tarrytown, NY, USA) with the accelerating voltage of 75 kV. The hydrodynamic size and surface charges of NPs in the aqueous solution were measured based on dynamic light scattering (DLS) using a Malvern Zetasizer Nano ZS (Malvern, Worcs, UK) before and after functionalization with tumor targeting ligand Tf. The fluorescent spectra of dye-labeled NPs were collected using a fluorescent spectrophotometer (Agilent Cary Eclipse, Santa Clara, CA, USA). The concentration of the protein ligands (Tf) conjugated on the surface of uIONPs or IONPs was quantified using a micro BCA protein assay kit following vendor's instructions (Thermo Fisher Scientific, Waltham, MA, USA) to determine the number of ligands conjugated on the NP surface. The IONP concentration (mmol/mL) was calculated based on the iron concentration of solution with the assumption that uIONPs and IONPs were spherical with a bulk magnetite density of 5.18 g/cm^3^ 44. The number of antibodies conjugated to each uIONP was determined by the ratio of the protein concentration (mmol/mL) of the antibody-conjugated uIONP solution to the uIONP concentration (mmol/mL).

### *In vitro* and* ex vivo* evaluation of TfR targeting by FITC-Tf-uIONPs

To evaluate active targeting FITC-Tf-uIONPs and passive targeting TRITC-uIONPs* in vitro*, TfR over-expressed 4T1 mouse mammary tumor cell lines were used. 4T1 breast cancer cell lines were purchased from ATCC (Manassas, VA, USA) and cultured following vendor's instructions. 4T1 cells (with 10% FBS containing DMEM medium) were seeded in 8-well chamber slides at the concentration of 50,000 cells per well for testing cell uptake of uIONPs or IONPs. After being attached to form a confluent monolayer, the medium was replaced with that containing FITC-Tf-uIONPs or TRITC-uIONPs at the concentration of 0.2 mg Fe/mL, and incubated for 4 hours at 37 °C. The cell monolayer was then rinsed with PBS, and fixed with 4% paraformaldehyde. Finally, 4',6-diamidino-2-phenylindole (DAPI) reagent was applied to stain cell nuclei. Uptake of fluorescent dye-labeled uIONPs by 4T1 cells was observed under a confocal microscope (Zeiss LSM510, Jena, Germany).

To further confirm the specific interaction between FITC-Tf-uIONPand TfR on 4T1 cells, a blocking experiment was conducted. Specifically, 200 µL of free Tf (1 mg/mL in FBS-supplemented RPMI-1640 medium) was first introduced to 4T1 cells to block free TfR presented on cell surfaces. After 1 hour incubation, the cell medium was replaced with that containing FITC-Tf-uIONPs at the concentration of 0.2 mg Fe/mL. After 4 hour incubation, cells were rinsed with PBS, fixed with 4% paraformaldehyde, and stained with DAPI for confocal fluorescence imaging.

For *ex vivo* validation of the targeting effect of FITC-Tf-uIONPs, tumor-targeting FITC-Tf-uIONPs and non-targeting TRITC-uIONPs were co-cultured with tumor slices collected from mice bearing 4T1 tumors. Briefly, an equal amount (0.1 mg Fe per uIONP sample or 0.5 mg/ml) of FITC-Tf-uIONPs and TRITC-uIONPs were added onto tumor slices which were then incubated at 4 °C overnight. After co-cultured with two types of uIONPs, tumor slices were first washed three times with PBS, and then mounted onto glass slides using the ProLong gold antifade mountant containing DAPI reagents for cell nucleus staining. The fluorescent images were then taken with a confocal fluorescence microscopy (Zeiss LSM510, Jena, Germany).

### Cellular toxicity of uIONPs

The cytotoxicity of FITC-Tf-uIONPs and TRITC-uIONPs was measured by the 3-(4, 5-Dimethylthiazol-2-yl)-2, 5-diphenyltetrazolium bromide (MTT) assay. Briefly, 4T1 breast cancer cells were seeded in the 96-well microplate at the concentration of 5,000 cells per well. After 24h recovery (37 °C), the medium was replaced with that containing uIONPs at various concentrations (0, 8, 16, 32, 63, 130, and 250 μg Fe/mL). After 24 hour incubation (37 °C), the cell medium was discarded and carefully washed with 1X PBS several times. Subsequently, 20 μL of MTT solution (5 mg/mL) was added into each well. After being incubated for 3 hours, the resulting purple formazan was mixed into DMSO. Finally, the absorbance of all samples was measured with microplate reader (Biotech Synergy2) at 540 nm. The cell viability (%) was calculated by dividing the absorbance of uIONP-treated cells to that of the control cells without uIONP treatment.

### *In vitro* macrophage uptake of FITC-labeled NPs

FITC-labeled IONPs with different size (3, 10, 20 and 30 nm) were used to study the size-dependent difference in cell uptake of NPs by macrophages. In brief, Raw264.7 macrophage cells purchased from ATCC (Manassas, VA, USA) were seeded in 8-well chamber slides with 10% FBS containing RPMI-1640 medium at the concentration of 10,000 cells per well. After a confluent monolayer of cells was formed, the cell medium was substituted with that containing FITC-IONPs of different sizes at the concentration of 0.2 mg Fe/mL, respectively. The mixtures were incubated at 37 °C for 4 hours. The cell monolayer was washed with PBS, and fixed with 4% paraformaldehyde. Finally, the cell nucleus was stained with DAPI reagent before observation under a confocal microscope (Zeiss LSM510, Jena, Germany).

### Animal tumor model and co-injection of different types of IONPs

All animal experiments were carried out following a protocol approved by Institutional Animal Care and Use Committee (IACUC). The orthotopic 4T1 mouse mammary tumor model was established by injecting 2 × 10^6^ of 4T1 breast cancer cells into the mammary fat pad of 6 to 8 weeks old female Balb/c mice. Tumors were allowed to grow for 10-14 days before reaching a volume of approximately 100 mm^3^ for experiments.

To directly compare different targeting effects in intratumoral delivery and retention of NPs, we co-injected two different NPs pared together, *e.g.*, both active targeting and non-targeting NPs of the same size, into the same tumor-bearing mice. The co-injection approach ensures the same delivery route and physiological environment that both agents navigate through, thus effectively minimizing the inter-subject variations. Specifically, an equal dosage (10 mg Fe/kg) of FITC-Tf-uIONPs and TRITC-uIONPs (or FITC-Tf-IONPs and TRITC-IONPs) was intravenously co-injected into the tail veins of the same 4T1 tumor-bearing mice. After co-injection, mice were euthanized at 1, 3 and 24 hours (n= 3/group) to observe the accumulation and retention behavior differences between active targeting and non-targeting NPs using multiphoton imaging.

### *Ex vivo* multiphoton imaging of NPs in tumors

After collecting the tumors at each time point, which were then sliced into 2 mm thick blocks, a two-photon microscope (Zeiss LSM 710, Germany) with a Chameleon Ti:sapphire NIR-tunable laser (Coherent Inc., USA) was used to scan the fresh tumor blocks to acquire fluorescent images from ROIs of the tumor tissue blocks at the wavelength of 880 nm. Multiphoton microscopy imaging of the tumor blocks enables in-depth measurement and comparison of intratumoral distribution of paired two types of NPs in the same ROI, with better spatial information and resolution on both 2D and 3D view 38-39. In addition, tumor stroma collagen fibers were visualized using second harmonic generation (SHG, filter sets BP 520-560) which presented as bright signals in multiphoton images. Two sets of multiphoton images were sequentially obtained from the same ROI with three signals, including the second harmonic generation (SHG, the gray fluorescent signal collected with filter sets BP 520-560), and FITC-Tf-uIONPs (green fluorescent signal collected with filter sets BP 500-550 red); and SHG and TRITC-uIONPs (red fluorescent signal collected with filter sets BP 565-610). The resolution for all multiphoton images was 512 × 512 pixels (NND) and a scan speed of 2.00 μs per pixel was used. The 3D reconstruction of SHG and FITC-Tf-uIONPs, and SHG and TRITC-uIONPs was completed by Bitplane's Imaris 3D software (Zürich, Switzerland).

### Confocal fluorescence imaging

To prepare tumor slices for *ex vivo* imaging experiments, the entire tumors collected at different time points after co-injection of NPs were immersed into optimal cutting temperature (OCT) compound, followed with being snap-frozen in liquid nitrogen. Frozen tumor tissue sections (7 µm thick) were examined using confocal fluorescent imaging for localizing intratumoral distribution of fluorescent dye-labeled NPs. Briefly, tumor tissue sections containing NPs were first washed three times with 1X PBS, and then mounted onto glass slides using ProLong gold antifade mountant, which contained DAPI for cell nucleus staining. Macrophages were stained by anti-CD68 antibody with fluor647 tagging. Fluorescent images were then taken with a confocal microscopy (Zeiss LSM510, Jena, Germany).

### Prussian blue staining for iron in tumors

Prussian blue staining was applied to qualitatively detect the uptake of uIONPs in tumor tissues. Briefly, the tumor tissue sections were fixed with ice cold acetone and then rinsed with 1X PBS, and then incubated with a freshly-prepared mixture of potassium ferrocyanide (II) trihydrate (4% (v/v)) and hydrochloride acid (4% (v/v)) for 2 hours, followed with 30 min culturing with nuclear fast red solution for cell nuclei and cytoplasm staining. Tumor tissue sections were dehydrated in a graded series of ethanol (75, 90 and 100%), and mounted for examination with light microscopy.

### Hematoxylin and eosin stain (H&E) staining

H&E staining was performed to determine the morphology of tumor tissue sections. Briefly, the tumor tissue sections were first rinsed with 1X PBS, and then cultured with 400 µL of hematoxylin 2 solution for 2 mins. Subsequently, the tumor tissue section was counterstained by 400 µL of eosin Y solution for 30 seconds. The staining differentiation was carried out by dipping the slide in a pre-mixed acidic alcohol solution (1% (v/v) HCl in 70% ethanol). Finally, H&E stained slides were step-wisely dehydrated in ethanol (95 and 100%) and xylene, and mounted for microscopic examination.

### Quantifications of NP uptake images based on pixel intensity-based threshold criterion

Pixel intensity analysis was performed by measuring the pixel intensity between purest blackness (0) and purest whiteness (255) and further filtering pixels with those values. After segmenting pixels of SHG and FITC-Tf-NPs, and SHG and TRITC-NPs based on their color and pixel intensity threshold, averaged pixel values of each color component were calculated for quantifying FITC or TRITC labeled NPs in each image slice and at different time points. Pixel values of FITC or TRITC and pixel ratios of FITC over TRITC from image slices numbers were plotted for each time point for comparison of intratumoral accumulation of two different types of NPs and the contribution difference between active and passive targeting to tumoral delivery of NPs.

## Supplementary Material

Supplementary information and figures.

## Abbreviations

ATF: amino terminal fragment
DAPI: 4',6-diamidino-2-phenylindole
DLS: dynamic light scattering
EDC: 1-ethyl-3-(3-dimethylaminopropyl)-carbodiimide
EPR: enhanced permeability and retention
FITC: fluorescein isothiocyanate
H&E: hematoxylin and eosin
IONPs: iron oxide nanoparticles
MRI: magnetic resonance imaging
NPs: nanoparticles
NHS: N-hydroxysuccinimide
ROI: regions of interest
SHG: second harmonic generation
TAMs: tumor associated macrophages
TEM: transmission electron microscope
Tf: transferrin
TfR: transferrin receptor
TRITC: tetramethylrhodamine
uIONPs: ultrafine iron oxide nanoparticles
