# Supplementary material for: Probing and Enhancing Ligand-Mediated Active Targeting of Tumors Using Sub-5 nm Ultrafine Iron Oxide Nanoparticles
Source: Theranostics. 2020 Jan 22;10(6):2479–94. doi: 10.7150/thno.39560 (PMC7052897; doi:10.7150/thno.39560)
Supplement: Supplementary file 1 — Supplementary information and figures. [file thnov10p2479s1.pdf]

Probing and Enhancing Ligand-Mediated Active Targeting of Tumors Using Sub-5 nm Ultrafine Iron Oxide Nanoparticles

Yaolin Xu[1], Hui Wu[1], Jing Huang[1,2], Weiping Qian[3], Deborah E. Martinson[4], Bing Ji[1], Yuancheng Li[1], Yongqiang A. Wang[5], Lily Yang[3] and Hui Mao[1,]*

[1]Department of Radiology and Imaging Sciences, Emory University School of Medicine, Atlanta, Georgia, USA

[2]Boston Children's Hospital, Harvard Medical School, Boston, Massachusetts, USA

[3]Department of Surgery, Emory University School of Medicine, Atlanta, Georgia, USA

[4]Integrated Cellular Imaging Core, Emory University, Atlanta, Georgia, USA

[5]Ocean Nanotech, LLC, San Diego, California, USA

Corresponding Author: Hui Mao, Department of Radiology and Imaging Sciences, Emory University, 1841 Clifton Road, Atlanta, Georgia 30329, USA, E-mail: hmao@emory.edu.

SUPPORTING INFORMATION

Materials and chemicals. All the chemicals were used without further purification. Ferric nitrite ($FeNO_3 \cdot 9H_2O$, 98%), sodium oleate (NaOA, 97%), hexane, ethanol, 1-octadecene (90%), chloroform, dimethylformamide (DMF, 99%), D-(+)-glucose, dimethyl sulfoxide (DMSO, 90%), ammonium hydroxide (NH_4OH, ACS grade), sodium bicarbonate ($NaHCO_3$) buffer (0.1 M, pH=8.5), fluorescein isothiocyanate (FITC), tetramethylrhodamine (TRITC), 1-ethyl-3-(3-dimethylaminopropyl)carbodiimide (EDC), n-hydroxysuccinimide (Sulfo-NHS), 4′,6-diamidino-2-phenylindole (DAPI), paraformalin, potassium ferrocyanide (II) trihydrate ($K_4Fe(CN)_6$), nuclear fast red solution, hematoxylin and eosin Y solution, and optimal cutting temperature compound (OCT) were purchased from Sigma-Aldrich (St. Louis, MO, USA). Oleic-acid coated iron oxide nanoparticles (IONPs) with an averaged core size of 30 nm, activation buffer (pH = 5.5) and coupling buffer (pH = 8.5) were purchased from Ocean Nanotech LLC (San Diego, CA, USA). Phosphate-buffered saline (PBS), fetal bovine serum (FBS), transferrin (Tf), RPMI-1640 medium, Dulbecco's Modified Eagle Medium (DMEM), trypsin-EDTA, penicillin-streptomycin solution, and 3-(4,5-dimethylthiazol-2-yl)-2,5-diphenyltetrazolium bromide (MTT) kit were purchased from Sigma-Aldrich (St. Louis, MO, USA). Amicon Ultra-4 centrifugal filters (50 kDa) were purchased from Millipore (Burlington, MA, USA). Balb/c mice were ordered from Harlan Laboratories (Indianapolis, IN, USA).

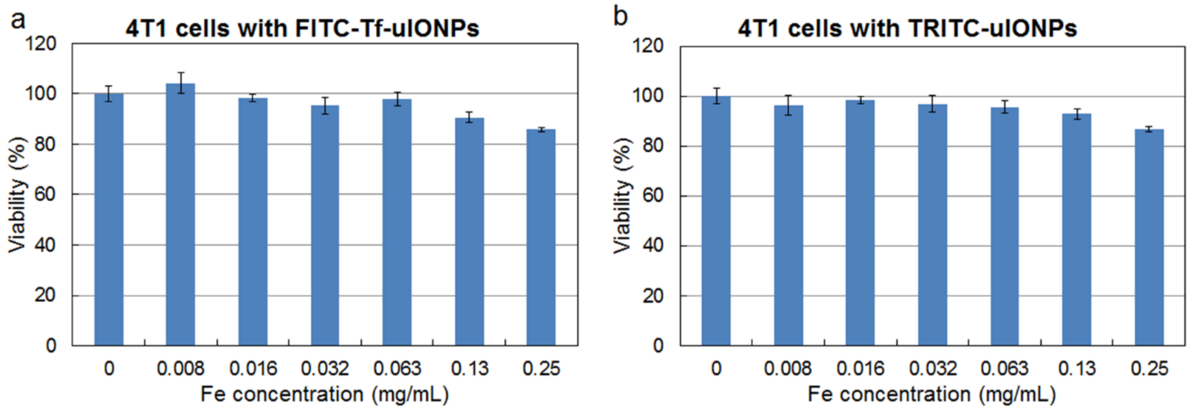

Figure S1. Cell viability studies of 4T1 breast cancer cells treated with (**a**) FITC-Tf-uIONPs and (**b**) TRITC-uIONPs;

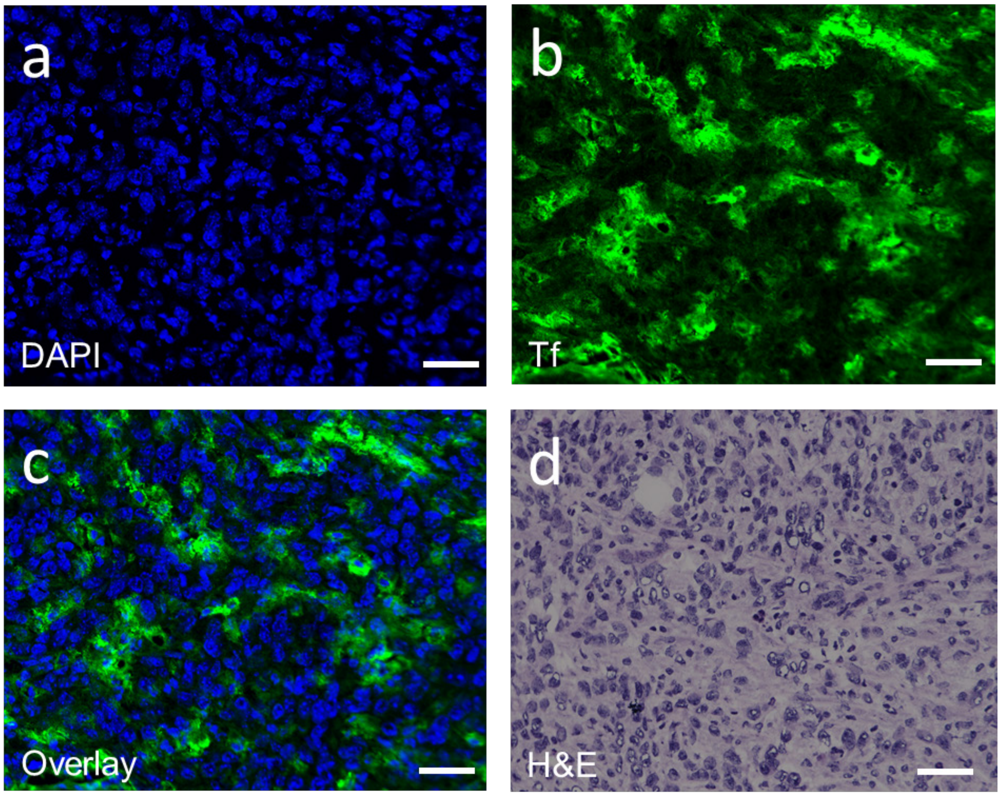

Figure S2. Confocal fluorescence images of a selected 4T1 tumor section co-stained with FITC labeled Tf. **a**: DAPI for nuclei, **b**: Tf; **c**: merged image with DAPI and Tf, (**d**) histological H&E staining from a tumor section adjacent to the section used for fluorescence imaging. The scale bar for all images is 100 μm.

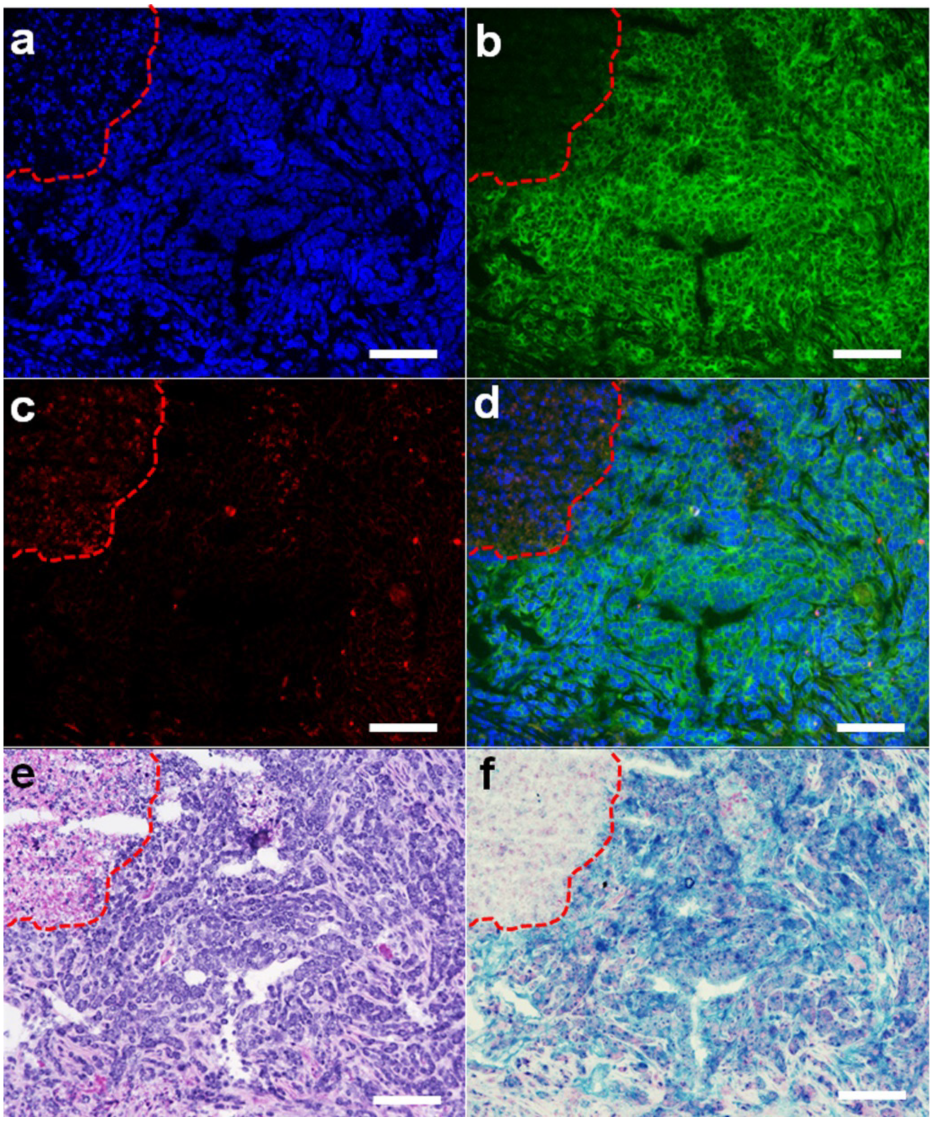

Figure S3. Confocal fluorescence images of a selected tumor section co-stained with FITC-Tf-uIONPs and TRITC-uIONPs. **a**: DAPI for nuclei, **b**: FITC-Tf-uIONPs (green), **c**: TRITC-uIONPs (red), and **d**: merged image (DAPI + FITC-Tf-uIONPs + TRITC-uIONPs); (**e**) histological H&E staining from a tumor section adjacent to the section used for fluorescence imaging. The dashed red lines outline the tumor regions with high cellularity (mainly co-localized with active targeting FITC-Tf-uIONPs) from the tumor stromal regions (mainly co-localized with non-targeting TRITC-uIONPs); (**f**) Prussian blue staining for iron on the same tumor section used for fluorescence imaging. The scale bar for all images is 200 μm.

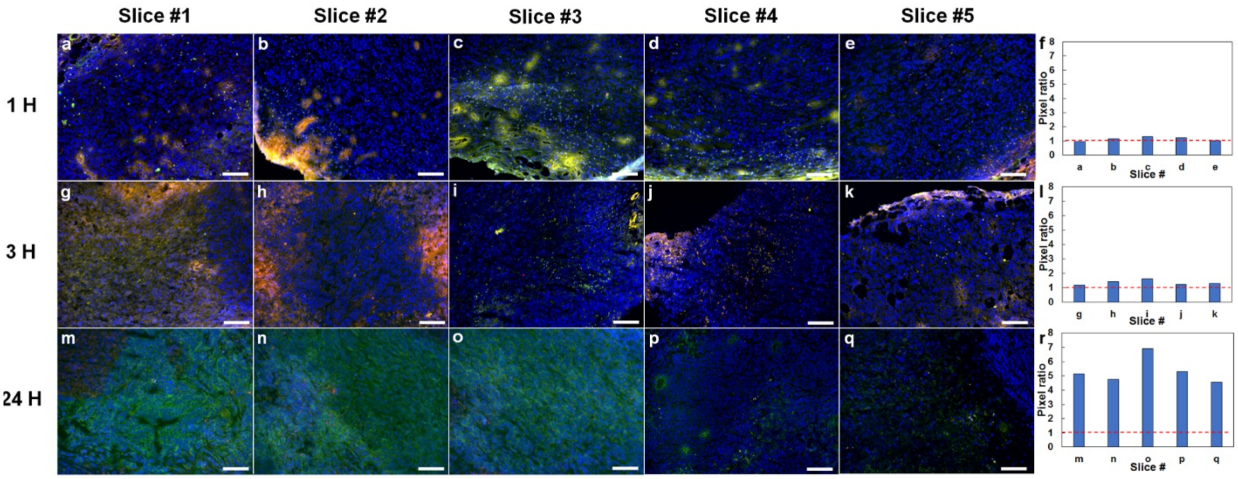

Figure S4. Merged confocal fluorescence images (DAPI + FITC-Tf-uIONPs + TRITC-uIONPs) of selected tumor slices collected from mice receiving co-injection of active-targeting FITC-Tf-uIONPs and non-targeting TRITC-uIONPs with the core size of 3 nm. Images were taken at different time points after co-injection of FITC-Tf-uIONPs and TRITC-uIONPs (**a-e**: 1 hour; **g-k**: 3 hours; **m-q**: 24 hours). The pixels of different fluorescent signals, *i.e.,* FITC-Tf-uIONPs (green) and TRITC-uIONPs (red), were segmented from each image and counted using an in-house program. Pixel ratios, defined as the pixel intensity of FITC-Tf-uIONPs over pixel intensity of TRITC-uIONPs, are plotted to demonstrate the time-dependent change of the ratios between active-targeting FITC-Tf-uIONPs and non-targeting TRITC-uIONPs (**f**: 1 hour, **l**: 3 hours, and **r**: 24 hours). The scale bar for all images is 200 μm.

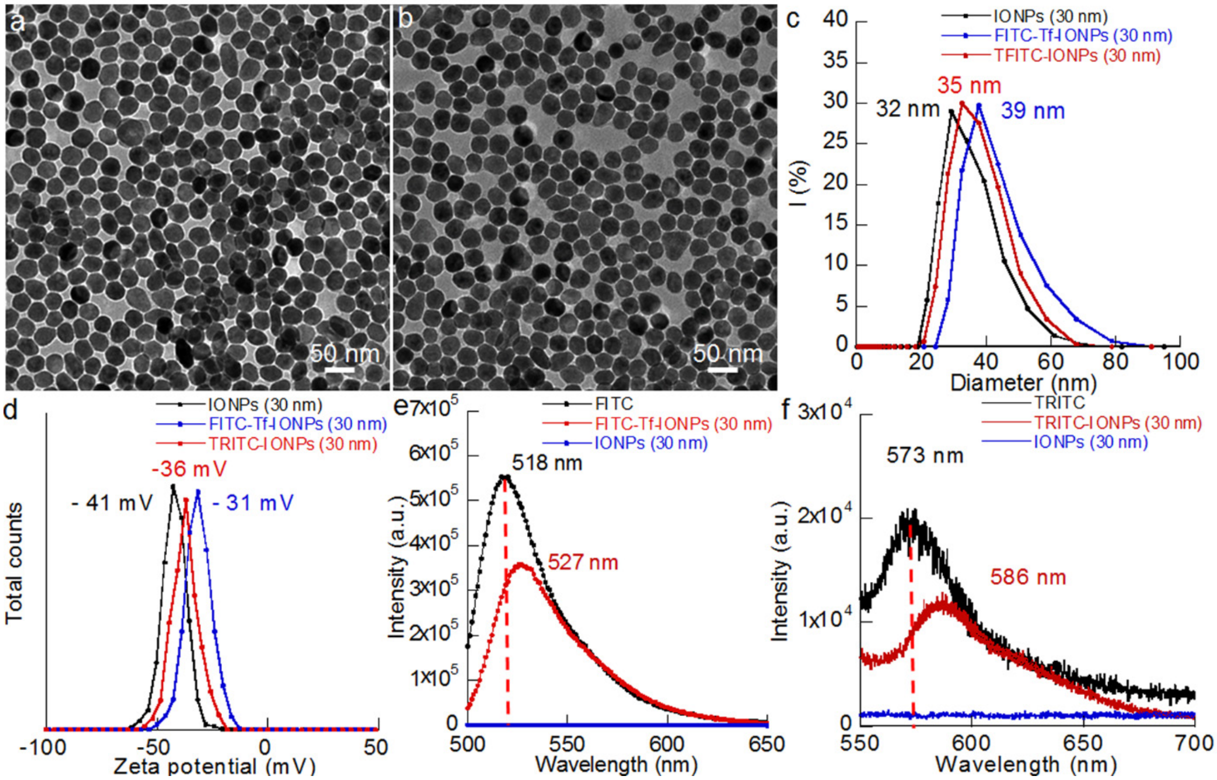

Figure S5. Characterizations of TRITC-IONPs and FITC-Tf-IONPs with the core size of 30 nm. TEM images of (**a**) TRITC-IONPs, and (**b**) FITC-Tf-IONPs; The size distribution (**c**) and zeta potential (**d**) of IONPs measured by DLS; Fluorescent emission spectra (**e**) and (**f**) of free dye, IONPs, and FITC-Tf or TRITC conjugated IONPs.

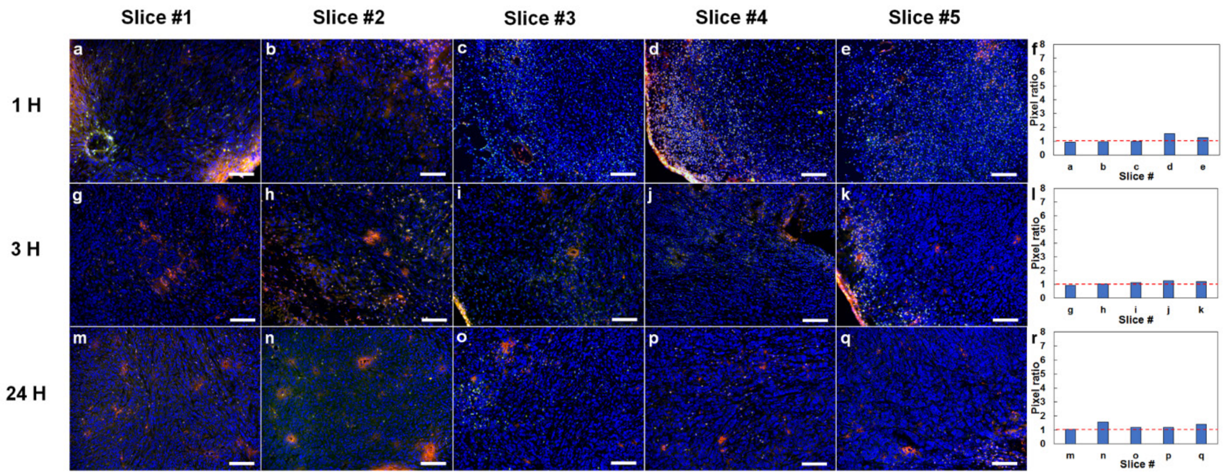

Figure S6. Merged fluorescent images of tumor sections collected from mice receiving co-injection of ligand conjugated active targeting FITC-Tf-IONPs and passive or non-targeting TRITC-IONPs with a 30 nm core size (**a-e**: 1 hour; **g-k**: 3 hours; **m-q**: 24 hours) and corresponding segmentation analysis (**f**: 1 hour, l: 3 hours, and **r**: 24 hours). The pixel ratio is defined as the pixel intensity of FITC-Tf-IONPs over pixel intensity of TRITC-IONPs. The scale bar for all images is 200 μm.

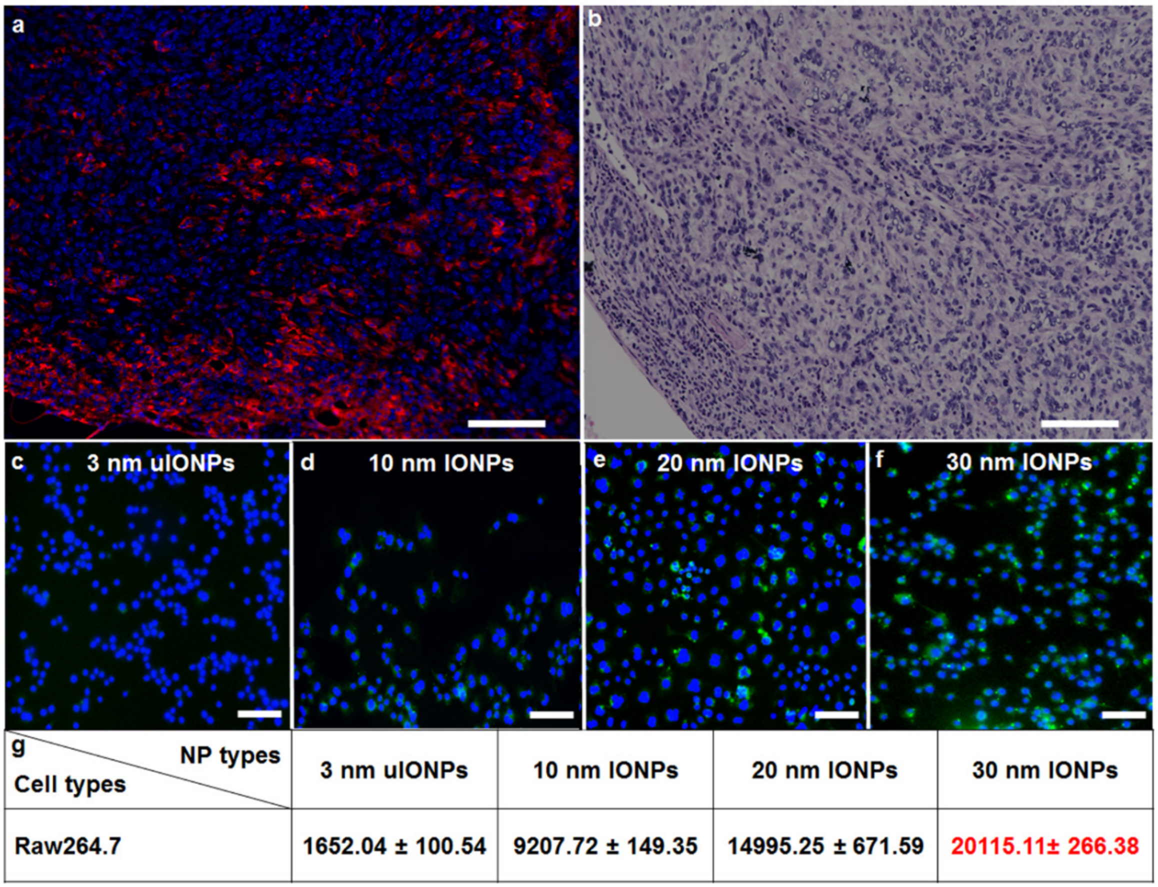

g Cell types \ NP types	3 nm uIONPs	10 nm IONPs	20 nm IONPs	30 nm IONPs
Raw264.7	1652.04 ± 100.54	9207.72 ± 149.35	14995.25 ± 671.59	20115.11± 266.38

Figure S7. Images of a selected 4T1 tumor tissue slice stained for CD68 macrophage (**a**) and corresponding H&E staining (**b**) (scale bar: 200 μm); Fluorescent images of mouse macrophage RAW264.7 cells treated with FITC labeled IONPs of different sizes (**c-f**) (scale bar: 20 μm), table summarizing pixel reading of different IONPs from fluorescent images to show the level of macrophage uptake (**g**).
